# Recent Developments in Deep‐Ultraviolet Flat Optics

**DOI:** 10.1002/nap2.70136

**Published:** 2026-05-26

**Authors:** Omar A. M. Abdelraouf, Tao Wang, Zeng Wang

**Affiliations:** ^1^ Institute of Materials Research and Engineering, Agency for Science, Technology, and Research (A*STAR) Singapore

## Abstract

Deep‐ultraviolet flat optics (DUVFO) is rapidly emerging as a foundational platform for next‐generation photonic technologies. Operating in the 200–300‐nm regime, DUVFO unlocks capabilities inaccessible to conventional refractive optics, enabling compact beam shaping, high‐NA (HNA) focusing, and efficient nonlinear interactions within ultrathin form factors. This review provides a comprehensive overview of the materials, device concepts, and system‐level innovations driving the shift from bulky, absorption‐limited DUV optics toward scalable, chip‐compatible flat‐optical architectures. We highlight the critical role of wide‐bandgap semiconductors and low‐loss metal oxides in overcoming intrinsic transparency and stability challenges; examine the evolution of linear and nonlinear DUV devices, from HNA metalenses and integrated spectroscopic modules to frequency conversion metasurfaces and chip‐scale light sources; and discuss their emerging impact in lithography, bioimaging, metrology, and nanofabrication. Finally, we outline the key bottlenecks in dispersion engineering, optical characterization, and wafer‐scale manufacturing, emphasizing the interdisciplinary advances required to transition DUVFO from laboratory demonstrations to manufacturable, high‐performance photonic platforms.

## Graphical abstract

Deep‐ultraviolet flat optics (DUVFO) is emerging as a key platform for next‐generation photonics, enabling advanced functionalities beyond conventional DUV optics. This review outlines materials, devices, and system advances, highlighting wide‐bandgap platforms, while identifying key challenges in dispersion control, characterization, and wafer‐scale manufacturing toward scalable, chip‐integrated DUV technologies.

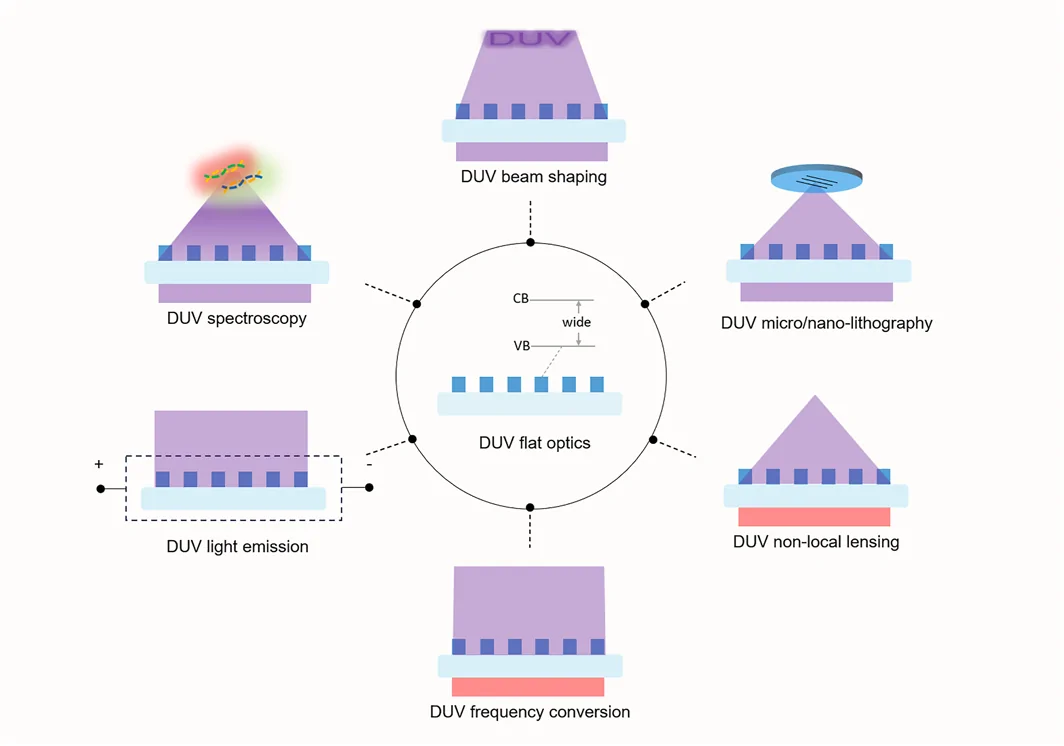

## 1 Introduction

### 1.1 Overview of DUVFO

DUVFO (*λ* = 200–300 nm) represents a frontier in the manipulation of light, offering critical solutions to longstanding challenges that have plagued traditional DUV optical systems. The imperative for DUVFO stems from fundamental physical limitations and technological bottlenecks that conventional refractive optics cannot effectively overcome. Traditional DUV optical systems face three critical pain points that severely constrain their practical implementation and integration into modern photonic circuits. First, efficiency limitations arise from material absorption and fabrication constraints [1]. At DUV wavelengths, most standard optical materials exhibit strong light absorption, rendering many conventional high‐index dielectrics unusable. Although transparent materials such as fused silica and calcium fluoride (CaF_2_) offer transparency, they lack the high refractive index contrast necessary for strong optical confinement, leading to weak light–matter interactions. Simultaneously, DUV sources, particularly those based on wide‐bandgap AlGaN semiconductors, historically suffer from low external quantum efficiencies (often < 10%) due to difficulties in p‐type doping, strong polarization‐induced electric fields, and high contact resistance [2, 3, 4]. Second, conventional systems suffer from excessive bulkiness and lack of integration. To compensate for low refractive indices and to minimize aberrations, traditional DUV systems rely on complex, heavy trains of curved refractive lenses. These systems are prohibitively expensive due to the several surface roughness reduction steps involved, occupy substantial footprints, and are fundamentally incompatible with the trend toward on‐chip miniaturization. This physical bulk prevents the deployment of DUV technology in portable sensing, integrated spectroscopy, and compact communication platforms. Third, lifetime and stability issues present significant hurdles. High‐photon‐energy DUV irradiation induces degradation in optical materials over time. This phenomenon is known as solarization, which alters refractive indices and increases absorption losses [5, 6]. Additionally, nanostructured devices face thermal instability and contamination‐induced degradation under high‐power operation, necessitating robust material selection and passivation strategies.

The fundamental appeal of flat optics lies in its ability to circumvent these limitations through subwavelength light manipulation. Nanophotonic structures, particularly dielectric metasurfaces and flat optics, exploit a variety of mechanisms of light–matter interactions to achieve efficient wavefront control, field enhancement, and light routing within a planar form factor. By engineering subwavelength features using wide‐bandgap semiconductors and low‐loss metal oxides such as aluminum nitride (AlN) and hafnium oxide (HfO_2_), DUVFO can realize effective refractive indices and phase modulations unattainable in bulk materials. This paradigm shift promises to transform DUV technology from laboratory‐scale equipment into accessible, miniaturized flat‐optical platforms [7, 8, 9].

### 1.2 Emerging Linear and Nonlinear DUVFO Applications

The manipulation of light by flat optics is fundamentally described by the generalized Snell’s law, which modifies classical refraction and reflection by introducing a spatially varying phase gradient at the interface. For a two‐dimensional metasurface situated in the *xy*‐plane, the modified momentum conservation requires that the transmitted and reflected wavevectors account for the artificial in‐plane phase gradients, $\frac{d\Phi }{dx}$ and $\frac{d\Phi }{dy}$, where *Φ* is the spatially varying phase profile imparted by the metasurface. This generalized refraction relation dictates that $\sin\left(\theta_{t}\right)n_{t}-\sin\left(\theta_{i}\right)n_{i}=\frac{1}{k_{0}}\frac{d\Phi }{dx}$, where *k*_0_ represents the free‐space wavenumber. This spatially varying phase is typically imparted through two primary mechanisms: the propagation phase, achieved by varying the geometric dimensions of dielectric resonators; and the Pancharatnam–Berry (PB) geometric phase, which relies on the spatial orientation of anisotropic meta‐atoms [10].

Building on these phase engineering principles, DUVFO has rapidly advanced in both linear and nonlinear regimes. In the linear regime, the field is currently witnessing a rapid expansion in free‐space flat optics for beam shaping, micro/nanofabrication, submicrometer‐resolution DUV spectroscopy, and active optoelectronic devices [11]. For example, the development of high‐numerical‐aperture (HNA) metalenses has revolutionized DUV imaging and lithography without the need for complex DUV objectives. Direct laser writing microscopy (DLWM) systems utilizing flat optics have demonstrated remarkable platform integration, enabling a direct way to characterize HNA without relying on an HNA DUV optical system [12]. Furthermore, the integration of nanostructures with active devices is gaining momentum. This includes the enhancement of light extraction in AlGaN nanowire LEDs through photonic crystal patterning and the exploration of tunable metasurfaces that can dynamically modulate DUV wavefronts, offering a pathway toward agile, reconfigurable DUV optical systems [2, 13].

In the nonlinear regime, compact light sources benefit significantly from the enhanced light confinement provided by nanostructures. Recent demonstrations of nanophotonic DUV third‐harmonic generation (THG) in crystalline silicon and dielectric metasurfaces have shown efficient frequency conversion from the near‐infrared (NIR) to the DUV region. The engineering of high‐*Q* nonlocal metalenses, particularly those supporting bound states in the continuum (BIC), enables strong local field enhancement, thereby boosting nonlinear conversion efficiency while simultaneously providing control over the far‐field wavefront of the generated harmonics. It paves the way for chip‐scale coherent DUV sources that overcome the phase‐matching limitations of traditional bulk crystals [14, 15, 16] (Figure 1).

**FIGURE 1:**
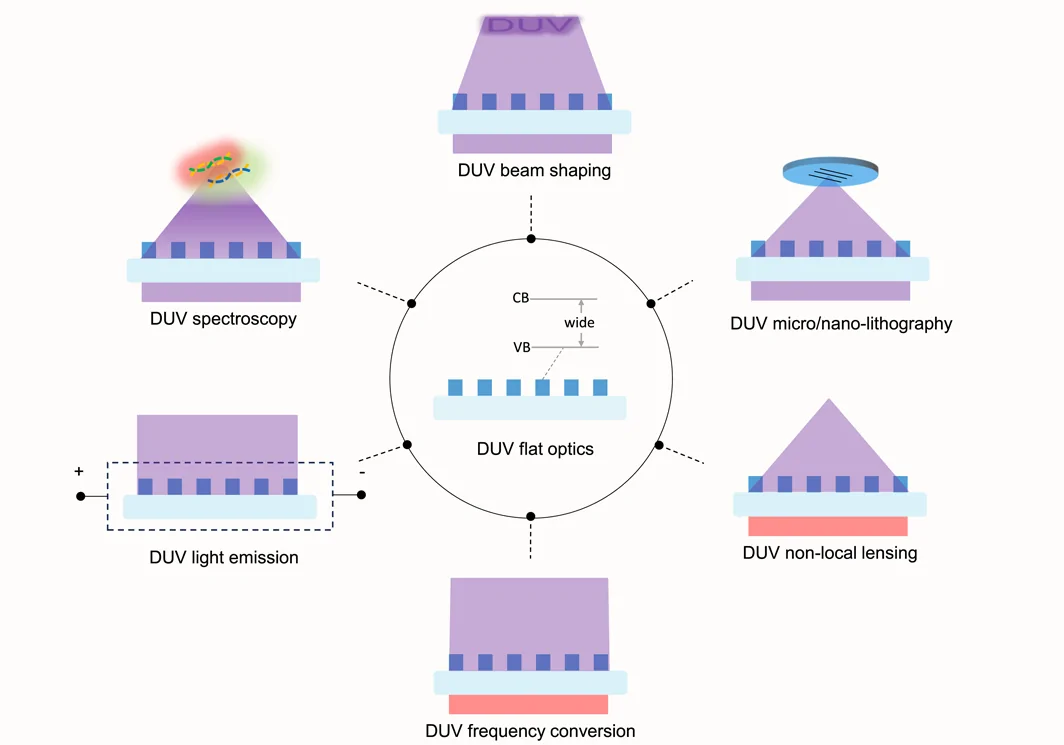
Overview of emerging applications of linear and nonlinear DUVFO.

### 1.3 Scope of the Review

Here, we present a focused examination of DUVFO, emphasizing the material foundations that fundamentally distinguish DUV metasurfaces from their visible and infrared counterparts. Because optical loss, bandgap constraints, and radiation stability are far more stringent in the DUV, we concentrate on wide‐bandgap platforms, examining their optical properties, bandgap engineering strategies, and stability under DUV irradiation. Design aspects are discussed only briefly, as most metasurface design principles such as phase control, unit‐cell engineering, and inverse design remain largely consistent across wavelength ranges and do not exhibit major conceptual deviations in the DUV. Furthermore, we address the critical challenges and practical constraints associated with the wafer‐scale manufacturing and integration of DUVFO.

We then highlight emerging linear and nonlinear applications uniquely enabled by DUVFO, including HNA focusing, lithography, subwavelength spectroscopy, far‐UVC imaging, and nonlinear frequency conversion. These examples demonstrate both the replacement of bulky refractive components and the creation of new DUV functionalities that were previously inaccessible.

Finally, we outline key challenges and perspectives. First, realizing broadband or achromatic operation remains difficult due to the intrinsically large material dispersion in the DUV, which severely limits phase compensation bandwidth. Second, optical characterization for high‐numerical‐aperture structures is technically demanding owing to the lack of commercial DUV objectives, limited detector sensitivity, and strong absorption in air and optics. Third, achieving wafer‐level (8 inch and 12 inch) manufacturing requires breakthroughs in high‐quality epitaxy, uniformity control, phase profile sampling optimization versus critical dimension, and low‐defect processing compatible with industry‐scale substrates. Addressing these challenges will be essential for transitioning DUVFO from laboratory demonstrations to robust, manufacturable photonic platforms. Collectively, this review positions DUVFO as a rapidly advancing technology capable of reshaping next‐generation photonic, spectroscopic, and nanofabrication systems.

## 2 Materials for DUVFO

### 2.1 Wide‐Bandgap Semiconductors

Wide‐bandgap semiconductors such as AlN, AlGaN, and β‐Ga_2_O_3_ are foundational for DUVFO, providing the necessary transparency, stability, and low‐loss characteristics for high‐efficiency DUV metasurfaces and integrated photonic components. Recent progress in epitaxy and optimized buffer‐layer or superlattice designs has significantly reduced defect densities and surface roughness, directly enhancing DUV optical performance in these materials. Moving forward, scalable deposition on large‐area glass or Si wafers will be crucial for translating these material advances into manufacturable DUV metasurfaces, flat‐optical elements, and photonic chips. Together, these developments position wide‐bandgap platforms to enable next‐generation DUV meta‐optical systems, driving the field toward fully integrated, wafer‐scale DUV photonics.

#### 2.1.1 Optical Properties and Significance

Bandgap engineering serves as a foundational tool for DUVFO and optoelectronics, allowing the precise tailoring of a material’s absorption edge and refractive index. By strategically alloying material compositions, designers can mitigate intrinsic absorption while maximizing the refractive index contrast necessary for strong Mie resonances [17]. Furthermore, in DUV light‐emitting devices, bandgap engineering is imperative for enhancing the external quantum efficiency (EQE). Tailored multiple‐quantum‐well (MQW) designs and superlattice structures effectively reduce defect‐assisted nonradiative recombination and alleviate the strong polarization‐induced quantum‐confined Stark effect (QCSE) [18]. Wide‐bandgap semiconductors such as gallium oxide (*β*‐Ga_2_O_3_, *E*_g_ ≈ 4.9 eV) and aluminum gallium nitride (Al_*x*_Ga_1‐*x*_N, *E*_g_ = 3.4–6.2 eV) exhibit a unique combination of large bandgaps, strong electronic and thermal stability, and tunable optical properties, making them pivotal for DUV optoelectronics, including light detection, emitters, and communication devices [19, 20, 21, 22, 23, 24, 25, 26, 27]. Aluminum nitride (AlN) is an ultrawide‐bandgap material (*E*_g_ ≈ 6.2 eV) with superior solar‐blind behavior, high breakdown field (12–15 MV/cm), and excellent thermal conductivity (∼340 W/m·K), ideal for DUV operation [28]. AlN and Al‐rich AlGaN alloys are crucial for DUV LEDs, laser diodes, and far‐UVC sterilization systems. Alloying GaN with Al enables emission across the UV‐A, UV‐B, and UV‐C regions, while maintaining GaN's advantages of radiation hardness and high‐speed operation [26, 27]. *β*‐Ga_2_O_3_ complements the nitride family with its ∼4.8 eV bandgap, high breakdown field, and strong chemical stability. Its intrinsic solar blindness allows low‐noise DUV detection, and its compatibility with scalable fabrication makes it promising for robust DUV detection and power devices [29]. Low‐dimensional forms, such as nanowires and 2D sheets, further improve integration and response speed for next‐generation compact DUV sensors.

#### 2.1.2 Epitaxial Growth and Crystal Quality Control

Achieving high‐performance DUV nanophotonic devices requires epitaxial films with excellent crystallinity and smooth morphology. Sputtered dielectric films offer a fast, flexible, and highly scalable manufacturing route. However, they typically exhibit amorphous or polycrystalline domains with elevated surface roughness and defect densities, which can be problematic for high‐efficiency DUV phase modulation. Conversely, advanced epitaxial growth techniques minimize atomic‐level imperfections, providing the atomically smooth interfaces and crystalline integrity required to suppress scattering and nonradiative recombination. Although epitaxial growth delivers superior optical film quality, it is more costly and strictly limited by substrate lattice‐matching requirements. Therefore, the choice between sputtering and epitaxy depends on the specific device requirements, balancing the need for ultralow optical loss against fabrication flexibility and cost [30, 31]. According to established surface scattering models, such as the Payne–Lacey (PL) model, scattering loss scales inversely with the wavelength and is proportional to the fourth power of the free‐space wavenumber [32]. Specifically, the scattering attenuation coefficient can be modeled such that loss scales with the square of the root‐mean‐square (RMS) surface roughness. At DUV wavelengths (200–300 nm), the short wavelength drastically amplifies the scattering coefficient. Consequently, even subnanometer roughness introduces catastrophic scattering losses that degrade the quality factor of resonant meta‐atoms. Sputtered dielectric films typically exhibit amorphous or polycrystalline domains with elevated surface roughness and defect densities. In contrast, advanced epitaxial growth techniques minimize atomic‐level imperfections, providing the atomically smooth interfaces and crystalline integrity required to suppress scattering and nonradiative recombination [31]. AlGaN and AlN grown on sapphire, SiC, or Si face large lattice mismatches (∼13% for AlN/sapphire), resulting in high threading dislocation densities (∼10^10^ cm^−2^) and nonradiative centers that degrade optical efficiency [33, 34, 35, 36, 37]. Compositional inhomogeneity, strain, and rough surface morphology further reduce near‐band‐edge emission and optical quality. For *β*‐Ga_2_O_3_, heteroepitaxy on sapphire or other substrates introduces phase instability and rough surface morphology unless growth temperature, precursor chemistry, and nucleation are tightly controlled [38, 39, 40, 41]. Crystalline defects and surface roughness contribute to scattering and absorption losses that scale inversely with wavelength, making DUV devices highly sensitive to surface quality [42, 43, 44].

#### 2.1.3 Buffer‐Layer Engineering for Crystal Quality Enhancement

Buffer‐layer engineering and heterostructure design play key roles in improving epitaxial quality. For AlGaN/AlN, AlN buffer layers and patterned sapphire substrates effectively reduce dislocation densities below 10^8^ cm^−2^ and achieve RMS roughness below 1 nm [45]. For *β*‐Ga_2_O_3_, introducing graded *β*‐(Al_*x*_Ga_1‐*x*_)_2_O_3_ layers or low‐temperature buffers enhances 2D growth and phase stability [40]. Incorporating heterostructures and superlattices can overcome doping limitations and enable tunable optical and electronic properties. For instance, (AlN)_5_/(GaN)_1_ superlattices improve Mg‐doping activation in Al‐rich AlGaN, whereas p‐NiO/n‐*β*‐Ga_2_O_3_ heterojunctions achieve bipolar‐like operation despite the absence of p‐type *β*‐Ga_2_O_3_. Alloying *β*‐Ga_2_O_3_ with Sc further extends its bandgap to ∼5.25 eV [46, 47].

### 2.2 Low‐Loss Oxides and Fluorides

Metal oxides with ultrawide bandgaps represent a critical class of materials for DUV photonic applications, offering exceptional transparency in the deep‐ultraviolet regime combined with remarkable thermal, mechanical, and chemical stability. Among these, hafnium oxide (HfO_2_), zirconium oxide (ZrO_2_), aluminum oxide (Al_2_O_3_), and strontium stannate (SrSnO_3_) have recently emerged as promising candidates for advanced nanophotonic devices operating at wavelengths below 300 nm [48, 49, 50].

#### 2.2.1 Hafnium Oxide (HfO_2_)

Hafnium oxide stands out as a premier dielectric material for DUV applications due to its ultrawide bandgap (5.7–6.3 eV depending on crystalline phase), high refractive index (1.98–2.14 in the visible‐to‐near‐UV range), excellent thermal stability, and compatibility with silicon‐based fabrication processes [51]. Hafnium zirconium oxide solid solutions (Hf_1−*x*_Zr_*x*_O_2_) offer additional tunability, with compositional control enabling bandgap engineering from 5.17 to 5.81 eV across the composition range. Notably, these mixed oxides exhibit composition‐dependent second‐harmonic generation that scales with ferroelectric remanent polarization, demonstrating their potential as silicon‐compatible nonlinear optical materials for integrated DUV photonics. The high thermal conductivity of HfO_2_ (exceeding 1 W/m·K) and exceptional chemical resistance make it suitable for harsh‐environment DUV optics, whereas its high dielectric constant (*k* ∼ 20–25) enables advanced gate dielectric and capacitor applications in optoelectronic circuits [52].

#### 2.2.2 Zirconium Oxide (ZrO_2_)

Zirconium oxide complements HfO_2_ with a slightly wider bandgap (5.1–5.5 eV in the monoclinic phase) and excellent DUV transparency, extending to wavelengths below 240 nm. ZrO_2_ exhibits remarkable phase tunability: Monoclinic, tetragonal, and cubic polymorphs can be stabilized through compositional doping or thermal processing, each offering distinct optical and structural properties. Zr‐doped SnO_2_ alloys have demonstrated bandgap engineering from the UV‐B region into the deep ultraviolet, with optical response tunable across DUV wavelengths through compositional control [53]. The material’s high refractive index (*n* ∼ 2.1–2.2), combined with low optical loss and good film‐forming properties via solution processing or atomic layer deposition, positions ZrO_2_ as an attractive platform for DUV waveguides, resonators, and optical coatings.

#### 2.2.3 Aluminum Oxide (Al_2_O_3_)

Aluminum oxide, particularly in its sapphire single‐crystal form, represents the gold standard for DUV transparency with a bandgap of approximately 8.8 eV and transmission extending to wavelengths below 150 nm. The material exhibits a broad transparency window from deep UV to mid‐infrared, extremely low propagation losses (below 0.1–1 dB/cm for amorphous thin films), high thermal conductivity (∼30–40 W/m·K for sapphire), and exceptional mechanical hardness. These properties make Al_2_O_3_ the preferred substrate material for AlGaN‐based DUV LEDs and photodetectors, whereas thin‐film Al_2_O_3_ deposited via atomic layer deposition or reactive magnetron sputtering enables low‐loss integrated photonic waveguides and optical coatings. The high refractive index contrast relative to silica cladding (*n* ∼ 1.65 for amorphous Al_2_O_3_ at 638 nm) facilitates tight mode confinement in waveguide structures while maintaining broad transparency from DUV through visible to mid‐IR [54].

#### 2.2.4 Strontium Stannate (SrSnO_3_)

Beyond the traditional HfO_2_, ZrO_2_, and Al_2_O_3_ oxides, emerging wide‐bandgap oxides extend DUV photonic capabilities. Strontium stannate (SrSnO_3_) represents a breakthrough as a deep‐ultraviolet transparent conducting oxide, exhibiting 85% optical transparency at a 300‐nm wavelength. Heterostructure engineering of SrSnO_3_ has enabled the realization of fully DUV‐transparent thin‐film transistors with all layers such as electrodes, gate dielectrics, and substrates while maintaining > 75% transmittance at 300 nm, opening pathways for invisible electronics operating in the solar‐blind UV regime [48]. The versatility of metal oxides for DUV nanophotonics stems from their unique combination of ultrawide bandgaps, high refractive indices enabling strong optical confinement, excellent thermal management properties, compatibility with wafer‐scale deposition techniques, and the ability to engineer optical and electronic properties through compositional alloying, phase control, and heterostructure design.

#### 2.2.5 Silicon Dioxide (SiO_2_)

Silicon dioxide remains the cornerstone low‐index dielectric for DUV optics and nanophotonic platforms, owing to its ultrahigh bandgap (∼9.0 eV for stoichiometric SiO_2_) and exceptional optical transparency extending below 160 nm. Amorphous and fused silica variants exhibit refractive indices ranging from 1.46 to 1.55 in the visible–UV spectrum, providing a crucial refractive index contrast when paired with high‐index oxides. SiO_2_ features extremely low optical absorption and scattering losses, enabling its use as cladding and spacer layers in DUV waveguides, antireflective coatings, and multilayer interference filters. Its low thermal expansion, outstanding chemical durability, and compatibility with silicon wafers facilitate integration with complementary photonic materials such as HfO_2_, Al_2_O_3_, and ZrO_2_ in multilayer metasurface architectures [5]. In DUVFO, SiO_2_ nanostructures serve as robust low‐index scaffolds that stabilize high‐aspect‐ratio metasurface elements while maintaining minimal parasitic absorption, supporting phase control and beam shaping down to sub‐200‐nm wavelengths [55].

#### 2.2.6 Calcium Fluoride (CaF_2_)

Calcium fluoride exhibits outstanding transparency from the vacuum ultraviolet (∼130 nm) through the infrared (∼9 μm) due to its ultrawide bandgap (∼12.1 eV) and low phonon absorption. With a refractive index of approximately 1.43 at 248 nm, CaF_2_ offers low dispersion and minimal birefringence, ensuring excellent chromatic performance across broad UV ranges [56]. Its cubic crystal symmetry supports isotropic optical properties, which is critical for polarization‐preserving DUV applications. In nanophotonics, CaF_2_ serves as an invaluable substrate for epitaxial film growth and as a low‐loss platform for UV photonic resonators and dielectric metasurfaces [57]. Owing to its radiation hardness, high laser‐damage threshold, and excellent environmental resilience, CaF_2_ is extensively employed in high‐power DUV lithography optics, window materials for UV laser systems, and as a low‐index pairing layer in refractive or hybrid DUV meta‐optical [58].

#### 2.2.7 Niobium Pentoxide (Nb_2_O_5_)

Niobium pentoxide represents a versatile high‐index dielectric (*n* = 2.2–2.4 at 400 nm) with a bandgap ranging from 3.5 to 4.2 eV, depending on stoichiometry and phase composition. Although its transparency onset lies at longer wavelengths than those of other wide‐bandgap oxides, careful stoichiometric control and oxygen‐rich processing extend usable transmission into the ultraviolet region. Its high refractive index, low absorption, and amorphous film quality produced via atomic layer deposition or reactive sputtering make Nb_2_O_5_ a strong candidate for all‐dielectric metasurfaces requiring enhanced phase manipulation capabilities at short wavelengths [45]. Furthermore, Nb_2_O_5_'s intrinsic nonlinear optical response opens prospects for reconfigurable nonlinear photonic devices [59].

#### 2.2.8 Magnesium Fluoride (MgF_2_)

Magnesium fluoride is among the most transparent crystalline materials available for deep‐ and vacuum‐ultraviolet applications, with a wide bandgap around 11.8 eV and transmission extending to 115 nm. Its moderate refractive index (*n* ≈ 1.37 at 250 nm) and low dispersion make it an ideal low‐index‐contrast material for multilayer DUV coatings, birefringent optical elements, and protective encapsulation layers [60]. MgF_2_'s low absorption coefficient and high laser‐damage threshold allow its use in extreme‐intensity UV lithography optics and polarizing components. In DUV nanophotonics, MgF_2_ serves as a low‐loss substrate or cladding for high‐index‐contrast metasurfaces, minimizing reflection losses while preserving efficient light confinement. Its wide phonon gap also ensures low nonlinear and thermal effects, making it valuable for precision wavelength control and phase‐stable operation in DUVFO and resonant photonic devices [61].

### 2.3 Advanced Wide‐Bandgap Semiconductors

Emerging wide‐bandgap semiconductors such as hexagonal boron nitride (hBN), diamond, and new 2D semiconductors are among the best candidates for DUV photonic devices. hBN stands out for its wide bandgap (∼6 eV), making it inherently transparent down to about 200 nm, with recent reports highlighting its use in DUV optoelectronics, waveguides, and nonlinear devices. hBN also exhibits strong excitonic features and high thermal stability, making it promising for on‐chip DUV photonics [62].

Furthermore, single‐crystal diamond (C) (*E*_g_ ∼ 5.5 eV) has garnered significant attention for DUV meta‐optical, photonic platforms, and high‐power devices due to its transparency down to ∼220 nm, high damage threshold, unparalleled mechanical hardness, chemical inertness, and the highest thermal conductivity of any bulk material [63, 64]. Recent fabrications of diamond metasurfaces have demonstrated highly efficient, broadband holographic wavefront shaping that maintains robust optical performance even at extreme temperatures (e.g., 500°C) and under harsh chemical exposure [63]. Furthermore, diamond nanostructures can sustain high‐Q Mie resonances in the UV, enabling Kerker‐like conditions that enhance the local density of optical chirality by more than three orders of magnitude for ultrasensitive chiral spectroscopy [65]. Beyond pristine diamond, heterostructures combining these wide‐bandgap semiconductors with 2D materials allow researchers to modulate electronic and optical behavior, control defect levels, and introduce entirely new nonlinear and quantum functionalities [66]. For instance, manipulating NV and SiV color centers in diamond under DUV light enables advanced quantum optics applications, whereas hybrid stacking of hBN with other 2D semiconductors provides tunable interfaces for DUV sensing and on‐chip photonic integration [67, 68, 69]. Ultimately, ongoing research to optimize material losses, engineer interfaces and buffer layers, and develop new materials remains critical for unlocking the full utility of these promising DUV platforms in high‐performance applications (Table 1).

**TABLE 1:** Summary of low‐loss materials for DUV applications.

Material	Bandgap (eV)	Refractive index (*n*)	Thermal conductivity (W/m·K)	Cutoff *λ* (nm)	Key advantages	Primary DUV applications
Al_*x*_Ga_1‐*x*_N	3.4–6.2	2.1–2.5	< 340	> 200 nm	Tunable bandgap; high electron mobility; efficient photoluminescence	DUV LEDs; optical communication; solar‐blind sensors
AlN	∼6.2	∼2.15	∼340	> 200 nm	Widest III‐nitride bandgap; high thermal conductivity; piezoelectric	Far‐UVC LEDs; flat optics; sterilization; SAW devices
*β*‐Ga_2_O_3_	4.5–4.9	1.9–2.1	10–27	> 240 nm	Ultrawide bandgap; radiation resistant; chemical stability	Solar‐blind detection; fast‐response imaging; power electronics
HfO_2_	5.7–6.3	1.98–2.14	1–2	> 190 nm	High‐*k* dielectric; CMOS‐compatible; phase‐tunable; NLO‐active	Gate dielectrics; optical coatings; DUV waveguides; ferroelectric photonics
ZrO_2_	5.1–5.5	2.1–2.2	2–3	> 240 nm	Phase‐tunable; excellent photocatalytic activity; high refractive index	DUV metasurfaces; optical coatings; photocatalysis; waveguides
Al_2_O_3_	∼8.8	1.76	30–40	> 140 nm	Broad UV transparency; exceptional thermal conductivity; high hardness	DUV substrates; low‐loss waveguides; optical windows; integrated photonics
SrSnO_3_	∼4.6	2.0–2.3	10–20	> 280 nm	DUV‐transparent conductor; high mobility; heterostructure‐compatible	DUV‐transparent electronics; invisible optoelectronics; conducting electrodes
hBN	∼6.0	1.8–2.1	∼400–600	> 200 nm	Ultrawide bandgap; high thermal conductivity; 2D layered structure	DUV emitters; quantum photonics; thermal management layers; 2D heterostructures
Diamond	∼5.5	2.41	∼2000	∼220 nm	Highest thermal conductivity; quantum defect centers; mechanical hardness	Quantum sensing; DUV optics; thermal management; color‐center photonics
SiO_2_	∼9.0	1.46–1.55	1.3–1.5	> 160 nm	Ultrawide bandgap; low optical loss; thermally and chemically stable	DUV claddings; interference coatings; metasurface scaffolds; integrated waveguides
CaF_2_	∼12.1	1.42–1.44	9–10	> 130 nm	Ultrawide transparency range; isotropic cubic crystal; radiation hard	DUV lenses; lithography optics; resonators; low‐index platforms
Nb_2_O_5_	3.5–4.2	2.2–2.4	1.5–2.5	> 290 nm	High refractive index; nonlinear optical activity; tunable stoichiometry	DUV metasurfaces; flat optics; tunable photonics; multilayer coatings
MgF_2_	∼11.8	1.36–1.39	19–23	> 115 nm	Broad DUV transparency; low refractive index; high laser‐damage threshold	DUV coatings; polarizers; protective layers; metasurface substrates

## 3 Applications

### 3.1 Linear Optics

#### 3.1.1 Flat Optics for DUV Far‐Field Beam Shaping and Manipulation

Extending meta‐optical devices into the DUV spectrum unlocks unique opportunities for miniaturized, high‐resolution photonic systems. At these short wavelengths, traditional bulky optics often suffer from high absorption, fabrication limitations, and alignment challenges, whereas metasurfaces offer planar, compact, and integrable alternatives with precise control over phase, amplitude, and polarization [70]. The material platform is critical for successful operation in the DUV regime. Conventional materials such as Si or TiO_2_ are limited by their narrow bandgaps and absorption below 300 nm [71]. Therefore, wide‐bandgap dielectrics such as AlN (*E*_g_ ≈ 6.0 eV), HfO_2_ (5.7 eV), and Si_3_N_4_ (5.0 eV) are increasingly used to ensure transparency and low loss down to 200 nm or even shorter wavelengths. These materials not only enable the formation of high‐aspect‐ratio nanostructures but also provide sufficiently high refractive indices (*n* > 2) to achieve full 2π phase modulation—an essential condition for efficient wavefront control (Figure 2).

**FIGURE 2:**
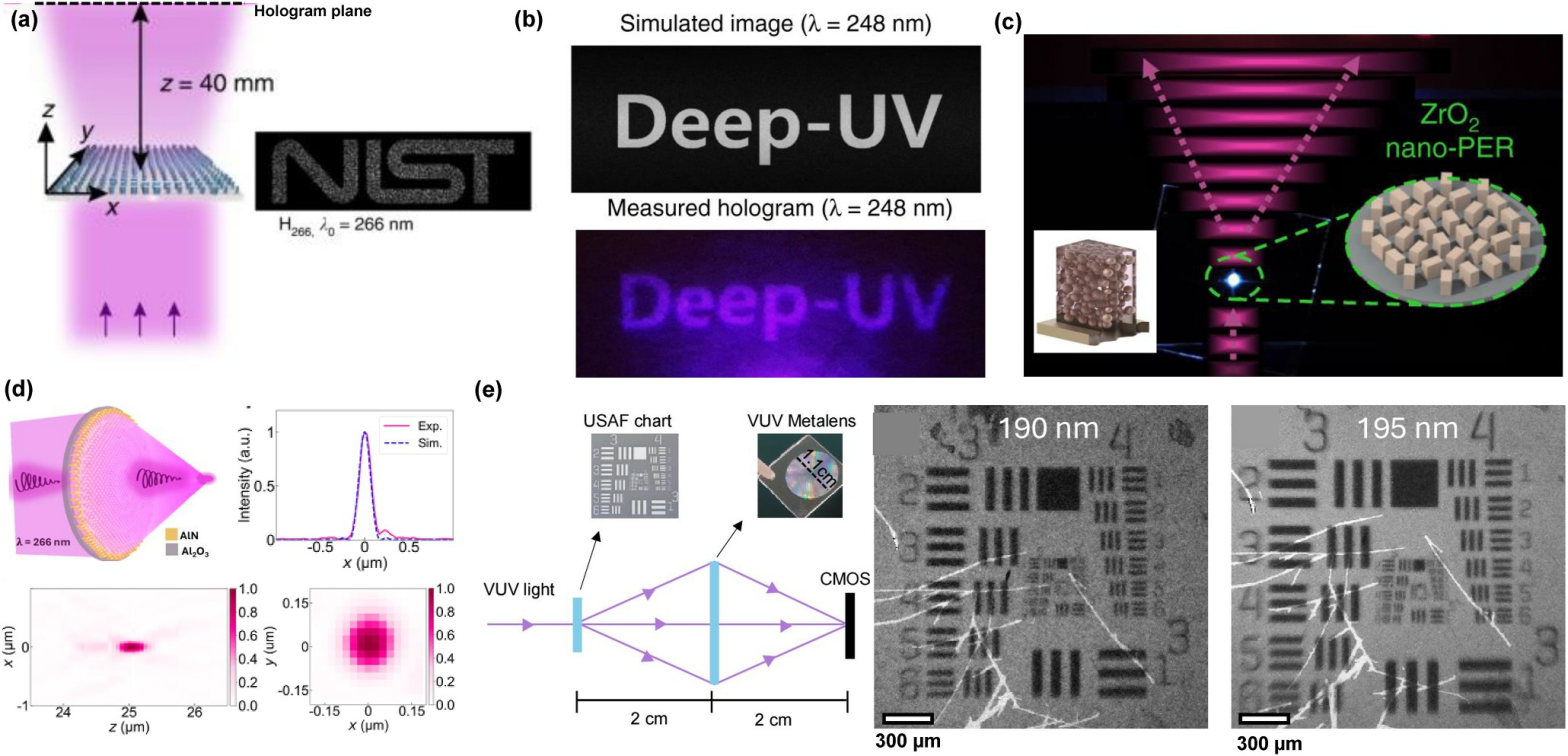
DUV meta‐optical devices. (a) Unit‐cell structure of a DUV meta‐hologram composed of HfO_2_ nanopillars for a meta‐hologram at 266 nm [49]. Reprinted with permission from Springer Nature (Copyright 2020). (b) DUV meta‐hologram based on ZrO_2_ [50]. Reprinted with permission from Springer Nature (Copyright 2023). (c) Nanoimprint ZrO_2_ nanoparticle‐embedded‐resin DUV meta‐optical. (d) AlN DUV metalens with high NA = 0.9 [12]. (e) Large‐area VUV metalens made from SiO_2_ nanopillars for imaging applications [55]. Reprinted with permission from Optica (Copyright 2025).

Recent demonstrations include meta‐holograms and metalenses fabricated from HfO_2_ nanopillars that achieve diffraction‐limited focusing (Figure 2a) [49]. Metasurface holography based on ZrO_2_ has further shown high‐fidelity image projection and vectorial encoding in the DUV band, reaching record diffraction efficiencies near 80% (Figure 2c) [50]. These results underscore the practical viability of metasurfaces in advanced UV lithography, microscopy, and optical encryption. To address dry etching fabrication challenges, researchers have adopted methods such as the resist‐based Damascene process combined with atomic layer deposition (ALD), enabling uniform deposition of nanostructured high‐bandgap materials with nanometer precision. However, the relatively low thermal conductivity of HfO_2_ limits its applicability in high‐fluence regimes. AlN, with its superior thermal conductivity (∼340 W/m·K), is a more suitable platform for high‐power DUV applications. A recent preprint reports significant advances in the design and nanofabrication of AlN‐based meta‐optical devices, enabling metalenses with numerical apertures approaching 0.9. These developments open a wide range of applications, including high‐resolution direct laser nanofabrication and spectroscopic imaging (Figure 2d) [12].

Besides, researchers have explored silica‐ and glass‐based materials as viable platforms for DUV and vacuum‐ultraviolet (VUV) wavefront control. For example, high‐efficiency glass‐based VUV metasurfaces have been successfully demonstrated (Figure 2e). However, utilizing silica presents unique fabrication challenges due to its relatively low refractive index in the DUV/VUV spectrum. To achieve the full 2π phase accumulation required for efficient wavefront shaping with a low‐index‐contrast material, the nanostructures must be designed with very high aspect ratios. Fabricating these tall, densely packed nanopillars is technologically demanding, as they are highly susceptible to structural collapse during wet processing and require highly selective, deep anisotropic etching profiles to maintain their geometric fidelity [55].

In stark contrast to the visible and near‐infrared regimes, where materials such as titanium dioxide (TiO_2_) and silicon nitride (Si_3_N_4_) have enabled broadband, full‐color achromatic metalenses through dispersion‐matched multilayers, extended group delay controls, and topological optimization [72, 73, 74], achieving broadband achromatic performance in the DUV remains fundamentally constrained. Wide‐bandgap semiconductors exhibit massive, nonlinear chromatic dispersion as wavelengths approach their electronic bandgaps [73]. This steep dispersion gradient demands exponentially growing group delay correction that single‐layer metasurfaces cannot physically provide without incurring prohibitive absorption losses.

#### 3.1.2 DUV‐Enhanced Microscopic and Spectroscopic Technologies

DUV light plays a transformative role in next‐generation bioimaging and sensing systems. Unlike visible light, DUV interacts strongly with biomolecular bonds, enabling inherently label‐free and chemically selective imaging. This makes DUV‐based techniques particularly attractive for clinical diagnostics, virology, and precision cytology. For instance, label‐free DUV microscopy has achieved high resolution, making it possible to visualize chromatin organization, intracellular structures, and even individual virus particles without fluorescent dyes [74]. In these systems, high‐resolution DUVFO is increasingly used to improve imaging uniformity and enhance focusing efficiency. Materials such as AlN, HfO_2_, and MgF_2_ provide the DUV transparency and fabrication compatibility needed to build integrated photonic structures. These materials have been used to demonstrate DUV high‐resolution microscopic imaging and high‐resolution spectroscopic PL imaging, as shown in Figure 3. As demonstrated in recent implementations of HNA DUV metalenses (Figure 2), achieving submicrometer photoluminescence spectroscopy fundamentally relies on HNA DUV metalenses. By precisely integrating an HNA DUV metalens to focus DUV light on aluminum gratings containing QDs, high‐resolution PL spectra were collected (Figure 3a). Moreover, a low‐NA DUV metalens (NA ≈ 0.2) has been used for DUV imaging of metal nanohole features down to 2 μm (Figure 3b).

**FIGURE 3:**
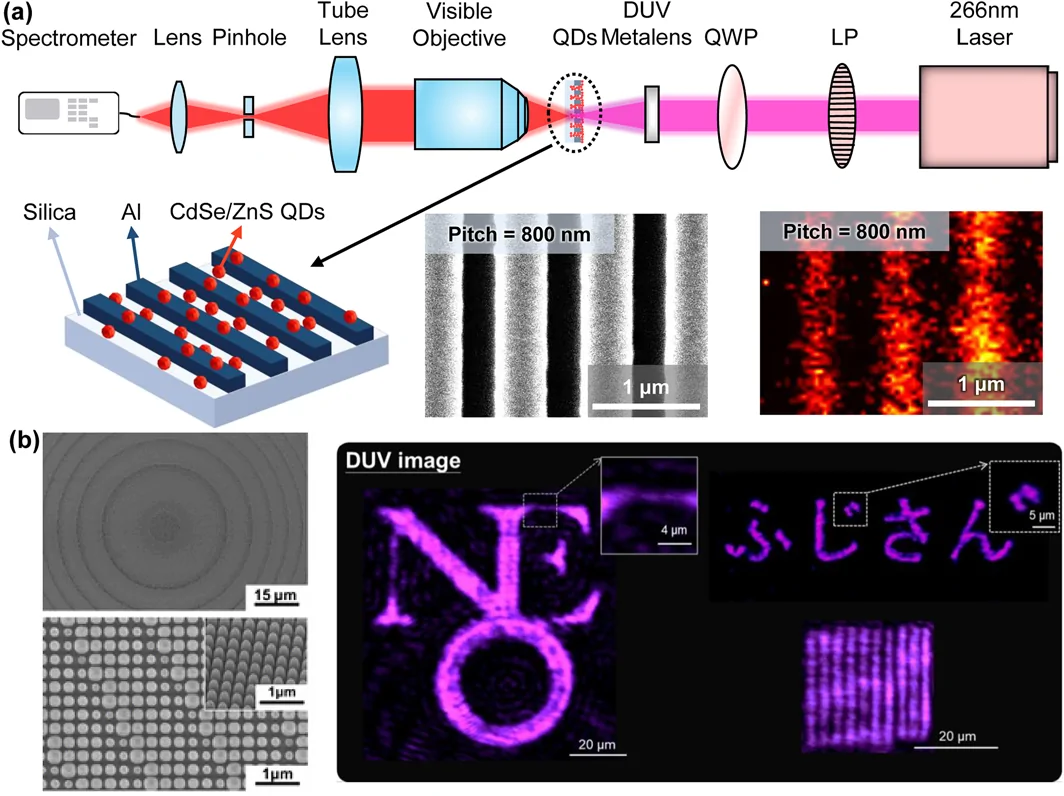
Nanophotonic DUV devices for imaging. (a) HNA DUV metalens enabling submicrometer PL spectroscopy (preprint [12]). (b) SEM image of a DUV polarization‐independent metalens [57] and the DUV imaging of micrometer hole structure in metal [75]. Reprinted with permission from American Chemical Society (Copyright 2025).

On the sensing front, DUV light offers unparalleled molecular specificity. It is especially effective in detecting biochemical fingerprints via absorption, fluorescence, or Raman scattering. Notably, deep‐UV resonance Raman spectroscopy (DUVRRS) can isolate vibrational signatures of DNA bases or protein structures with minimal background interference, paving the way for sensitive identification of pathogens, toxins, and disease markers. To miniaturize these capabilities, integrated DUV photodetectors are now being developed for sensing applications [76].

#### 3.1.3 DUVFO for Miniaturized Micro/Nanofabrication

DUVFO offers ultracompact footprints, high numerical apertures, and deterministic design freedom, making it highly attractive for portable direct laser writing (DLW) tools and other miniaturized micro/nanofabrication systems. By leveraging strong DUV photon–material interactions and subwavelength wavefront control, DUV metalenses can deliver tight focusing and flexible beam shaping without bulky refractive objectives. As shown in Figure 4a,b, Peng et al. [57] demonstrated this capability using an AlN metalens that enables ultrafast DLW with micrometer‐scale resolution, achieving localized ablation and microstructuring in a compact femtosecond‐DUV machining setup. Extending this further, Abdelraouf et al. [12] achieved 100 nm features in photolithographic DLW using an HNA DUV metalens and introduced DUV direct laser writing microscopy (DLWM) as a practical solution for characterizing HNA DUVFO, as displayed in Figure 4c,d. These advances confirm DUVFO as a key enabler for compact, high‐resolution, and scalable micro/nanofabrication technologies.

**FIGURE 4:**
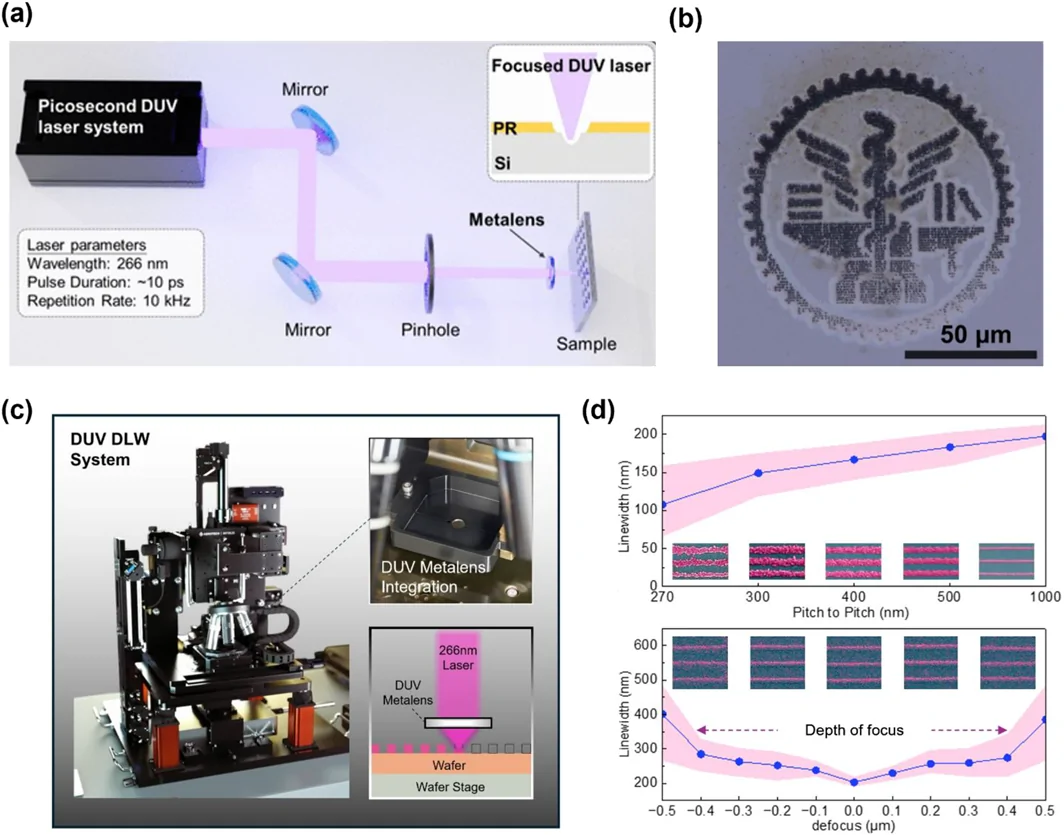
DUVFO for nanophotolithography. (a) Schematic of the experimental setup for ultrafast direct laser writing [75]. (b) Optical image of microfabrication features using the AlN metalens in (a). Reprinted with permission from American Chemical Society (Copyright 2025). (c) 3D schematic of fully automated direct laser writing based on a DUV metalens. (d) SEM images of nanofabricated grating lines with resolutions down to 150 nm [12].

#### 3.1.4 DUVFO for Light‐Emitting Devices

Integrating efficient DUV light sources directly onto photonic chips is a critical step toward miniaturized optoelectronic systems for sensing, diagnostics, and next‐generation spectroscopy. Among viable materials, III‐nitride semiconductors (AlGaN and AlN) are uniquely suited for emission in the 200–350‐nm range due to their tunable wide bandgaps and high stability. However, realizing high‐performance DUV emitters faces a fundamental “light extraction” bottleneck: As the aluminum content increases to reach shorter wavelengths (*λ* < 280 nm), the optical polarization switches from transverse electric (TE) to transverse magnetic (TM). TM‐polarized light propagates in the plane of the chip, making it notoriously difficult to extract from conventional planar surfaces [77, 78, 79].

To overcome these limitations, flat optical structures and nanostructuring have emerged as essential tools for wavefront engineering at the source. Planar LED architectures are increasingly incorporating dielectric metasurfaces and photonic crystals directly onto the emission surface to redirect in‐plane TM photons into the vertical extraction cone. Furthermore, the transition from planar films to AlGaN nanowire heterostructures represents a paradigm shift. Nanowires not only relax the strain induced by lattice mismatch, which reduces dislocation densities and nonradiative recombination, but also act as individual waveguides. By creating large‐area arrays of AlGaN nanowires, researchers have demonstrated significantly enhanced light extraction efficiencies compared to those of planar counterparts. These “3D” nanostructures effectively function as a flat optical layer, utilizing subwavelength scattering to optimize the far‐field emission pattern (Figure 5a) [80].

**FIGURE 5:**
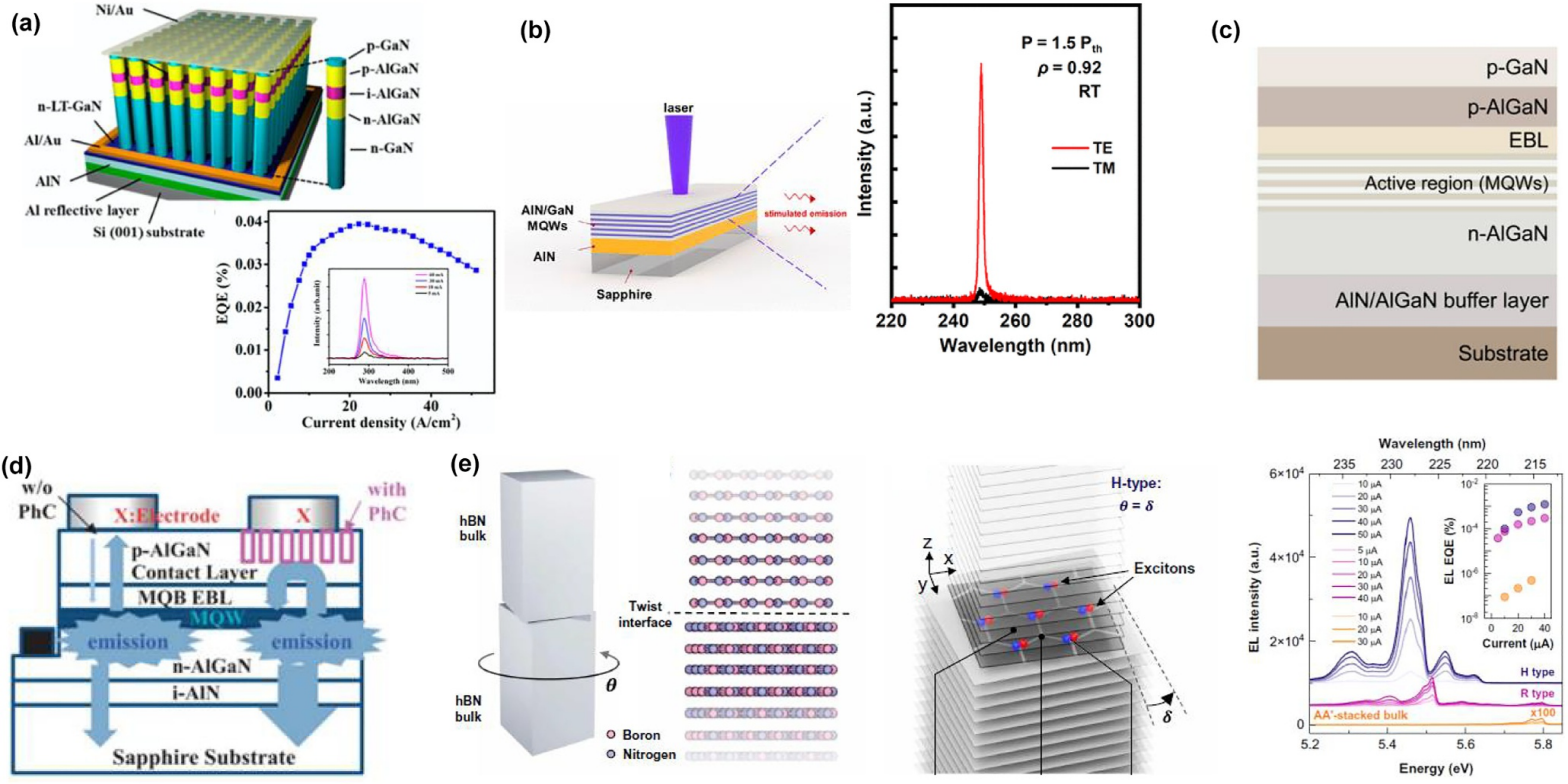
Nanophotonic enhancement of DUV light‐emitting devices. (a) Schematic of AlGaN nanowire LEDs, illustrating how nanostructuring reduces defect density and enhances the extraction of TM‐polarized light compared to planar films [80]. Reprinted with permission from MDPI (Copyright 2020). (b) Design of a sub‐250‐nm DUV laser based on AlGaN/AlN heterostructures. The structure utilizes high‐quality optical confinement within the active region to achieve lasing at wavelengths as short as 243 nm under optical pumping [81]. Reprinted with permission from American Chemical Society (Copyright 2019). 1D multiple quantum well DUV sources. (c) Epitaxial structure and energy band diagram of a typical AlGaN‐based DUV LED designed for sub‐250‐nm emission [82]. Reprinted with permission from MDPI (Copyright 2022). (d) Design schematics of DUV LEDs with photonic crystal layers, flip‐chip architectures, and tunnel junctions for enhanced light extraction and carrier transport [83]. Reprinted with permission from MDPI (Copyright 2022). (e) DUV optical exciton luminescence in hBN bulk twisted moiré quantum wells and electroluminescence (EL) of hBN moiré quantum wells with different moiré configurations compared with bulk hBN [84]. Reprinted with permission from American Association for the Advancement of Science (Copyright 2026).

In the lasing regime, flat optics enables the confinement and feedback necessary for coherent emission in compact footprints. A major challenge for DUV laser integration lies in designing high‐*Q*‐factor cavities that operate below 250 nm, where absorption in conventional materials is prohibitive. Recent breakthroughs have utilized AlGaN/AlN multiple quantum wells (MQWs) grown on AlN substrates to achieve sub‐250‐nm lasing. Notably, photopumped lasing at 243 nm has been demonstrated in these heterostructures, leveraging the high crystalline quality and strong optical confinement provided by the AlN platform. In these systems, cavity feedback is often provided by nanostructured elements, such as cleaved facets acting as Fabry–Pérot mirrors or lithographically defined distributed Bragg reflectors (DBRs) [81]. Advanced nanophotonic designs are pushing these capabilities further. Photonic crystal nanobeam cavities and vertical‐cavity surface‐emitting lasers (VCSELs) incorporating dielectric metasurface mirrors are being developed to reduce lasing thresholds. These flat optical cavities confine light in extremely small mode volumes, enhancing the Purcell factor and enabling stronger light–matter interactions. Efficient DUV nanolasers could be obtained using the abovementioned designs (Figure 5b).

In parallel, planar multiple‐quantum‐well DUV light sources have become essential tools in nanoscale imaging, biochemical diagnostics, and high‐resolution spectroscopy. Enabled by recent progress in nanophotonics and material engineering, these sources are increasingly replacing traditional mercury lamps and synchrotron systems due to their smaller footprints, better spectral tunability, and improved integration capabilities. Among them, AlGaN‐based light‐emitting devices offer precise control over emission wavelengths from 365 to 210 nm by adjusting the aluminum content. These devices have already demonstrated utility in fluorescence microscopy, nucleic acid detection, and environmental monitoring. However, pushing AlGaN emitters into the sub‐250‐nm DUV region poses significant challenges: p‐Type doping remains inefficient due to the high activation energy of Mg dopants in high‐Al‐content AlGaN; threading dislocations and point defects contribute to nonradiative recombination, limiting internal quantum efficiency (IQE); and light extraction efficiency is inherently low in this wavelength range. Addressing these issues requires improved epitaxial growth on lattice‐matched templates, defect passivation strategies, electron blocking layers, and optimized nanostructured outcoupling surfaces (Figure 5c) [82]. In parallel, cathodoluminescence (CL)‐based DUV light sources have emerged as promising alternatives, particularly in the 240–270‐nm range. These devices generate light by accelerating electrons onto phosphor or AlGaN targets, thereby avoiding the need for p‐type doping altogether. Recent CL chips exhibit compact dimensions (< 3 cm), high power conversion efficiency (> 4%), and superior ON/OFF response, making them ideal for applications such as disinfection, label‐free biomolecular detection, and portable analysis platforms (Figure 5d) [83].

Beyond conventional emission mechanisms, an emerging frontier in DUV light sources lies in the exploitation of moiré physics in van der Waals (vdW) materials. Recent breakthroughs have demonstrated the formation of moiré quantum wells (QWs) at the twisted homojunction interfaces of bulk hBN. These hBN moiré superlattices strongly confine charge carriers within atomically thin, periodic potential wells embedded in the 3D matrix. Despite hBN’s indirect bandgap, these localized excitons facilitate highly efficient phonon‐assisted radiative recombination, emitting intense far‐UVC luminescence (215–240 nm; Figure 5e). Remarkably, the deep‐ultraviolet photoluminescence from these twisted hBN homojunctions under both optical excitation and electrical carrier injection can exceed the intensity of state‐of‐the‐art AlGaN multiple QWs by more than an order of magnitude. By simply tuning the twist angle, the luminescence energy and efficiency can be widely modulated, establishing twisted vdW bulk materials as a highly promising, tunable platform for next‐generation, high‐efficiency DUV optoelectronics (Figure 5f) [84].

### 3.2 Nonlinear Optics

#### 3.2.1 Enhanced SHG and THG for Frequency Conversion

Second‐harmonic generation (SHG) and third‐harmonic generation (THG) are cornerstone mechanisms in nonlinear optics for generating DUV radiation from infrared or visible light [85]. Recent advances in nanophotonic engineering, including metasurfaces, plasmonic resonators, and high‐quality‐factor (high‐Q) nanocavities, have significantly enhanced harmonic conversion efficiency and directionality, making them viable for compact DUV sources in lithography, microscopy, and biochemical detection. To achieve effective frequency conversion in the DUV range, researchers commonly employ nonlinear materials with large second‐ or third‐order susceptibilities and broad bandgaps, such as lithium niobate (LiNbO_3_), beta‐barium borate (BBO), indium tin oxide (ITO), GaN, zinc oxide (ZnO), and AlN. Nanostructuring these materials into photonic crystal slabs, resonant metasurfaces, or quasi‐phase‐matched gratings can enhance local field confinement and compensate for phase mismatch, thus enabling more efficient harmonic extraction. Dielectric nanostructures further enhance field intensity through electromagnetic field localization. The nonlinear frequency conversion processes underscore the transition from conventional phase‐matched bulk crystals to subwavelength resonant nanophotonic cavities. The engineering of high‐quality‐factor (high‐Q) resonant flat optics, particularly those supporting bound states in the continuum (BIC), has drastically improved harmonic far‐field efficiency. Specifically, the utilization of modal phase‐matched dual BICs enables extraordinary local field enhancement, overcoming the phase‐matching limitations of traditional bulk crystals. Dual‐resonant architectures, in particular, enable modal phase matching and extreme local field confinement (Figure 6b) [14]. Among the candidate materials, crystalline silicon (c‐Si) has emerged as an unexpected yet powerful platform for DUV third‐harmonic generation (THG). Despite its intrinsic UV absorption, c‐Si can sustain strong near‐field intensities arising from interband plasmonic resonances, which amplify local electromagnetic hotspots within symmetry‐protected BIC arrays. This interband plasmonic character grants c‐Si metasurfaces exceptional nonlinear conversion efficiency and robust far‐field wavefront control, redefining material utilization strategies for DUV nanophotonics. Preliminary results demonstrate that through nonlocal phase‐gradient engineering and a high‐Q BIC metalens, precise control over the nonlinear light phase can be achieved, efficiently harnessing field localization for DUV harmonic generation (Figure 6d) [15]. Moreover, incorporating phase change materials in resonant DUV metasurfaces enables reconfigurable generation of DUV THG [16]. Moreover, research has demonstrated that ZnO nanodisks can generate 197 nm second‐harmonic generation by confining optical modes induced by a 394‐nm femtosecond laser (Figure 6c) [88]. High‐harmonic generation in bulk ZnO crystals and nanostructured ZnO flat optics has been developed to generate and focus DUV light using zone plate flat lenses [89, 90]. Toroidal dipole resonances in tailored metal‐dielectric metasurfaces have been shown to significantly boost 262 nm third‐harmonic generation under 785 nm pumping (Figure 6a) [86]. Moreover, broadband DUV light generation was demonstrated through amplification and concentration of surface currents in sub‐10‐nm‐gap gold plasmonic resonances [91]. These approaches leverage deep‐subwavelength resonances to enhance nonlinear polarization and frequency conversion efficiency.

**FIGURE 6:**
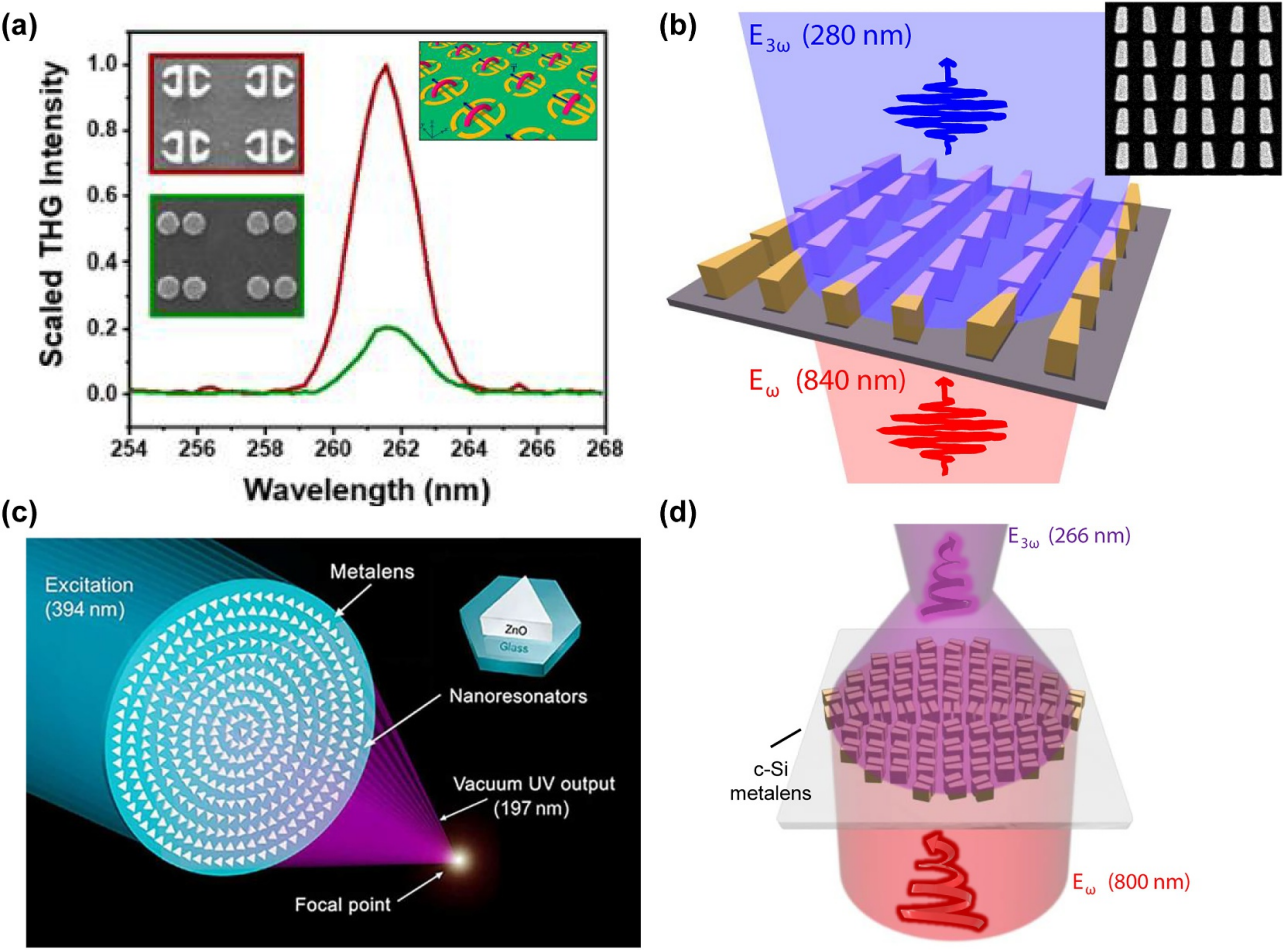
Dielectric metasurfaces for nonlinear frequency conversion. (a) Schematic of an ITO‐plasmonic metasurface enabling THG with large amplification in the DUV [86]. Reprinted with permission from American Chemical Society (Copyright 2025). (b) THG from a modal phase‐matched silicon BIC metasurface at a wavelength of 280 nm [14]. Reprinted with permission from American Chemical Society (Copyright 2024). (c) Illustration of a dielectric metasurface generating directional SHG signals through strong field enhancement and phase control [87]. Reprinted with permission from Science Advances (Copyright 2022). (d) Nonlocal BIC phase‐gradient metasurface for focusing DUV THG power [15].

## 4 Challenges and Opportunities

### 4.1 Achromatic and Broadband Operation Under Strong DUV Dispersion

Achieving broadband or achromatic performance in the DUV is fundamentally constrained by the strong material dispersion of wide‐bandgap materials, which severely limits phase compensation bandwidth. Because single‐layer metasurfaces cannot adequately correct the rapid index variation near the absorption edge, most DUV devices today remain narrowband. Possible solutions include multilayer or cascaded metasurfaces that distribute chromatic correction across several layers and hybrid diffractive–refractive architectures [92, 93, 94, 95]. In this context, utilizing AI‐assisted inverse design offers a powerful advantage by significantly accelerating chromatic aberration correction and enabling the seamless co‐optimization of phase, group delay, and dispersion across multiple geometries for complex, multifunctional flat‐optical devices [16, 96, 97]. Furthermore, the inclusion of physics‐informed neural networks empowers these models to explicitly adhere to foundational electromagnetic principles, allowing them to precisely suggest physically compatible designs and accurately predict optical performance [98]. Dispersion‐tailored high‐Q resonant meta‐atoms may further enable partial achromatization over tens of nanometers, offering a realistic pathway toward broadband DUV operation. Looking forward, the outlook for agentic AI is particularly promising; such autonomous systems could actively assist designers by intelligently navigating vast parameter spaces, dramatically streamlining the future development of broadband DUVFO (Figure 7).

**FIGURE 7:**
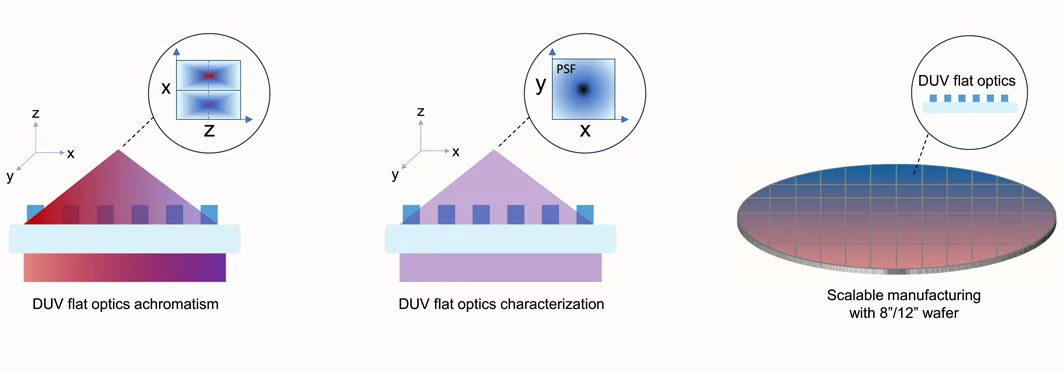
The challenges of DUVFO.

### 4.2 Optical Characterization of DUVFO

Optical characterization for high‐numerical‐aperture devices is a major challenge due to the lack of commercial HNA DUV objectives, limited detector sensitivity, and strong absorption of DUV light in air and available optics. These constraints hinder accurate measurement of focal spot size, efficiency, and aberrations. Emerging solutions include direct laser writing microscopy (DLWM) [11], which allows quantitative evaluation of HNA performance without requiring refractive DUV optics, as well as near‐field or knife‐edge scanning approaches that bypass HNA collection limitations [99]. Additional strategies such as fluorescence‐assisted probing and compact vacuum‐compatible test benches further improve measurement reliability and provide practical routes toward standardized DUV metasurface characterization.

### 4.3 Wafer‐Level (8″/12″) Manufacturing and Integration

Scaling DUV metasurfaces to 8‐inch and 12‐inch wafers is constrained by the need for extremely small critical dimensions (CDs), often < 100 nm, to achieve high NA and low loss at deep‐UV wavelengths [100]. These tight geometrical tolerances demand advanced wafer‐scale fabrication lines capable of high‐resolution patterning, uniform etching, and subnanometer surface control. A fundamental challenge arises from the optical requirements dictated by the Nyquist sampling theorem. To achieve a high numerical aperture (NA) and mitigate higher‐order diffraction anomalies, the metasurface unit‐cell pitch is typically designed to be smaller than *λ*/2NA. In the DUV regime (e.g., *λ* = 266 nm), targeting a high NA (e.g., NA > 0.9) pushes the theoretical pitch well below 150 nm, subsequently driving the critical dimensions of individual nanostructures into the sub‐50‐nm regime [99]. However, to further develop DUV metalens technology for scaled‐up manufacturing, such as utilizing immersion lithography for real‐world applications, a strategic compromise is often necessary. Unlike in the visible and infrared regimes, where larger wavelengths comfortably accommodate ideal subwavelength fabrication, the DUV regime requires balancing theoretical optical efficiency with the practical sampling of the phase mask. This involves adjusting the period and structure dimensions to meet the achievable critical dimensions (CDs) of current nanofabrication processes. Etching dense, high‐aspect‐ratio structures at deep‐nanometer scales uniformly across an 8‐ or 12‐inch wafer is hindered by microloading effects and mechanical pattern collapse. Therefore, co‐optimizing the phase sampling requirements for optical performance with the realistic CD limits of large‐scale nanofabrication stands as a defining consideration for the commercialization of DUVFO. At the manufacturing level, the realization of high‐performance DUVFO is fundamentally constrained by the stringent patterning requirements associated with sub‐100‐nm and increasingly sub‐50‐nm feature sizes. Achieving such critical dimensions requires advanced lithographic platforms, including 193 nm immersion lithography and, ultimately, extreme ultraviolet (EUV) lithography, both of which are built on 12‐inch wafer infrastructures. However, a significant gap currently exists in the availability and maturity of 12‐inch material platforms for wide‐bandgap semiconductors such as AlN. Scaling these materials to large‐area wafers remains challenging due to lattice mismatch, residual stress, wafer bowing, and defect propagation, which limit compatibility with industrial lithography and high‐throughput manufacturing. Bridging this gap requires coordinated advances in wafer‐scale epitaxy, substrate engineering, and process integration, together with the development of low‐damage and high‐fidelity pattern transfer techniques [101]. In parallel, the integration of inline, high‐resolution metrology will be essential to ensure uniformity, process stability, and yield. Addressing these challenges is critical for aligning DUVFO with established semiconductor manufacturing ecosystems and enabling its transition toward scalable, industry‐ready platforms.

## 5 Outlook and Conclusion

The field of deep‐ultraviolet flat optics has experienced remarkable growth over the past few years, driven by converging advances in materials science, nanofabrication techniques, and application demands across material metrology, biomedical imaging, and semiconductor manufacturing. This review has highlighted the emergence of mature technological platforms in the DUV regime.

Looking forward, several technological trends will likely shape future developments in DUVFO. The integration of AI approaches for device design and optimization has shown early promise, with ML techniques demonstrating the capability for addressing complex design challenges in DUV systems [16, 97, 98]. These approaches may accelerate the development timeline for next‐generation devices while improving performance predictability [102]. Material innovation remains a critical frontier, with organic molecular systems and metal‐organic frameworks offering potential advantages for specific applications through tailored optical properties and solution‐processable fabrication approaches. The development of hybrid organic–inorganic systems may enable new device architectures that combine the processing advantages of organic materials with the stability and performance characteristics of inorganic platforms [103].

The continued advancement of DUV nanophotonics requires sustained interdisciplinary collaboration across multiple scientific and engineering domains. The fundamental material challenges associated with high‐energy photon interactions demand close cooperation among materials scientists, solid‐state physicists, and device engineers to develop next‐generation platforms with improved stability and performance characteristics. Biomedical applications of DUV technologies necessitate strong partnerships between photonic researchers and biomedical scientists. The development of clinically viable DUV imaging systems requires a deep understanding of both the underlying photonic principles and the specific requirements of medical diagnostic applications. Similarly, the translation of research discoveries into commercial medical devices demands collaboration with regulatory agencies and healthcare providers to ensure that safety and efficacy standards are met [104]. Manufacturing scalability remains a critical challenge that requires sustained collaboration between research institutions and industrial partners. The transition from laboratory demonstrations to commercial deployment involves complex engineering challenges related to yield optimization, cost reduction, and reliability enhancement [105].

In summary, the field of DUVFO stands at a critical juncture where foundational scientific advances are beginning to enable transformative applications across multiple domains. The realization of this potential will require sustained interdisciplinary collaboration, strategic investment in research infrastructure, and careful attention to the broader implications of these powerful technologies. Through coordinated efforts across scientific, engineering, and policy communities, DUVFO can continue to drive innovation and enable breakthrough applications.

## Author Contributions

**Omar A. M. Abdelraouf:** visualization, writing – original draft, writing – review and editing, data curation, investigation, formal analysis, conceptualization. **Tao Wang:** writing – original draft. **Zeng Wang:** conceptualization, investigation, funding acquisition, writing – review and editing, writing – original draft, visualization, supervision, project administration, formal analysis.

## Conflicts of Interest

The authors declare no conflicts of interest.

## Data Availability

The data that support the findings of this study are available from the corresponding author upon reasonable request.
